# Cadmium Modifies the Cell Cycle and Apoptotic Profiles of Human Breast Cancer Cells Treated with 5-Fluorouracil

**DOI:** 10.3390/ijms140816600

**Published:** 2013-08-12

**Authors:** Yolande Asara, Juan A. Marchal, Esther Carrasco, Houria Boulaiz, Giuliana Solinas, Pasquale Bandiera, Maria A. Garcia, Cristiano Farace, Andrea Montella, Roberto Madeddu

**Affiliations:** 1 Department of Biomedical Sciences, University of Sassari, Viale San Pietro 43/B, Sassari 07100, Italy; E-Mails: yasara@uniss.it (Y.A.); gsolinas@uniss.it (G.S.); bandiera@uniss.it (P.B.); cfarace@uniss.it (C.F.); montella@uniss.it (A.M.); 2 Biopathology and Regenerative Medicine Institute (IBIMER), Centre for Biomedical Research, University of Granada, Granada E-18100, Spain; E-Mails: ecp_85@hotmail.com (E.C.); hboulaiz@ugr.es (H.B.); 3 Department of Human Anatomy and Embryology, Faculty of Medicine, University of Granada, Avda de Madrid s/n, Granada E-18100, Spain; 4 Oncology Department, University Hospital “Virgen de las Nieves”, Granada E-18100, Spain; E-Mail: mangelgarcia@ugr.es; 5 National Institut of Biostructures and Biosystems (INBB), viale delle Medaglie d’oro, Rome 00118, Italy

**Keywords:** cadmium, 5-fluorouracil, MCF-7, breast cancer

## Abstract

Industrialisation, the proximity of factories to cities, and human work activities have led to a disproportionate use of substances containing heavy metals, such as cadmium (Cd), which may have deleterious effects on human health. Carcinogenic effects of Cd and its relationship with breast cancer, among other tumours, have been reported. 5-Fluorouracil (5-FU) is a fluoropyrimidine anticancer drug used to treat solid tumours of the colon, breast, stomach, liver, and pancreas. The purpose of this work was to study the effects of Cd on cell cycle, apoptosis, and gene and protein expression in MCF-7 breast cancer cells treated with 5-FU. Cd altered the cell cycle profile, and its effects were greater when used either alone or in combination with 5-FU compared with 5-FU alone. Cd significantly suppressed apoptosis of MCF-7 cells pre-treated with 5-FU. Regarding gene and protein expression, *bcl2* expression was mainly upregulated by all treatments involving Cd. The expression of *caspase 8* and *caspase 9* was decreased by most of the treatments and at all times evaluated. *C-myc* expression was increased by all treatments involving Cd, especially 5-FU plus Cd at the half time of treatment. Cd plus 5-FU decreased *cyclin D1* and increased *cyclin A1* expression. In conclusion, our results indicate that exposure to Cd blocks the anticancer effects of 5-FU in MCF-7 cells. These results could have important clinical implications in patients treated with 5-FU-based therapies and who are exposed to high levels of Cd.

## 1. Introduction

Industrialisation and changes in human work activities have led to increased use of substances containing heavy metals, which has had significant economic and social consequences. These substances may also be harmful to human health. Cadmium (Cd) is a by-product of refining zinc and lead, being present in the raw ore, and is one of the most toxic heavy metals, because it is readily transferred via the air and water. Cd is also a notable constituent of cigarette smoke [1].

Cd can be absorbed by inhalation, ingestion, and direct contact with skin. It has a long half-life that ranged from 15 to 40 years, with a mean of about 20–25 years. Cd accumulates very easily in the liver and kidneys, and is considered one of the most dangerous heavy metals in humans because of its teratogenic and carcinogenic effects. These effects of Cd have been well established in humans and in animal models [2–5].

In people who are not occupationally exposed to heavy metals, the main route of exposure to Cd is via cigarette smoking [6,7], including passive smoking, as the metal is vaporized during combustion. Another common route of exposure is the consumption of contaminated food. Foods that often contain high levels of Cd include liver, offal, crustaceans, and molluscs. Nonetheless, around 80% of dietary Cd intake comes from cereals, potatoes and vegetables [8,9]. Occupational exposure to Cd mainly occurs in factories that dispose of batteries and accumulators that contain Cd, as well as in mines, and zinc processors [10]. In normal circumstances, very small amounts of Cd are absorbed by the human body, at a level that does not present a health concern. The toxicological properties of Cd are due to its chemical similarity to and ability to compete with zinc, an essential micronutrient for plants, animals, and humans [11]. It is thought that humans are constantly exposed to Cd present in the air and water, and that the daily intake in humans is between 0.5 and 1 μg [12].

The International Agency for Research on Cancer (IARC) classified Cd as a category 1 carcinogenic substance. Cd induces lung cancer, in particular, and recent experimental studies have demonstrated its close correlation with hematopoietic malignancies, as well as cancers of the bladder, stomach, and pancreas [4,5,13,14]. There is also increasing epidemiological evidence linking exposure to Cd with breast cancer [15,16]. The first study to reveal this link was a hypothesis-generating case—control study that examined the death certificates of over 33,000 deaths attributed to breast cancer and over 117,000 non-cancer deaths between 1984 and 1989 [17].

Breast cancer is the most common form of cancer in women in Western countries, and is the fifth most common cause of cancer deaths worldwide, after cancers of the lung, stomach, liver, and colon/rectum. The International Association of Cancer Registries (IACR) reported that breast cancer is the most common cancer in women, and accounts for 25% of all cancers in women. The breast cancer cell line MCF-7 was originally isolated in 1970 from a 69-year-old Caucasian American woman. The main features of MCF-7 cells include their luminal epithelial nature, their high invasive potential, the presence of oestrogen receptors (ER), and the marked proliferative response to oestrogen [18,19]. This cell line exhibits several characteristics of differentiated mammary epithelial cells, including the ability to metabolize oestradiol as a ligand for the cytoplasmic oestrogen receptor [20]. MCF-7 cells are frequently used as an experimental model to study the effects of pharmacological therapies [21–24]. The effects of Cd have been tested in many experimental studies using cell lines, including MCF-7 cells.

5-Fluorouracil (5-FU) is a fluoropyrimidine anticancer drug that disrupts cellular metabolism by inhibiting the synthesis of purines and pyrimidines, which disrupts DNA synthesis and RNA translation in target cells. In this way, 5-FU promotes cell death during cell division. In order to exert its cytotoxic activity, 5-FU must be enzymatically converted to a nucleotide by ribosylation and phosphorylation [25,26]. Approximately 90% of the administered dose of 5-FU is catabolized by dihydroprymidine dehydrogenase in the liver, peripheral blood mononuclear cells, intestinal mucosa, pancreas, lungs and kidneys; the remaining 10% is excreted unchanged in the urine [26]. 5-FU is an important chemotherapeutic drug and has been used for about 40 years. 5-FU is used in most of the standard chemotherapeutic protocols for solid cancers of the colon, breast, stomach, liver, and pancreas, among others. Moreover, 5-FU is able to induce differentiation in human tumour cells; however, it is highly toxic to both tumour cells and normal cells [27].

The *in vitro* models of human breast cancer using MCF-7 cells that were established in our previous study [28], have allowed us to investigate the mechanisms of Cd-related cytotoxicity associated with environmental exposure to Cd in contamined food, air, the working environment or cigarette smoking, and elucidate its impact on 5-FU chemotherapy of breast cancer [28,29]. We previously reported that Cd avoids the cytotoxic effects of 5-FU on breast cancer cells *in vitro* preventing the formation of lysosomes in the cytoplasm [28]. However, the underlying molecular mechanisms responsible for these effects were not determined. Therefore, the aim of this study was to analyse the biomolecular effects of Cd in 5-FU–treated breast cancer cells, with a particular focus on the cell cycle profile, apoptosis, and changes in gene and protein expression.

## 2. Results

### 2.1. Effect of Cd and 5-FU on Cell Cycle Analysis and Apoptosis

Cd induced marked changes in the cell cycle profile of MCF-7 cells. Our finding showed that Cd decreased the proportion of cells in the G0/G1 phase in comparison with control non-treated cells (M) over time. Thus, we observed 49% ± 1.19 *vs*. 61.1% ± 2.07, 66.9% ± 1.2 *vs*. 81.7% ± 2.88 and 60.5% ± 2.03 *vs*. 85.9% ± 3.21 in treated *versus* non-treated cells after 12 h, 24 h and 48 h, respectively (*p* = 0.0005). Moreover, an increased proportion of cells in the S phase were observed: 26.1% ± 0.56/21.7% ± 1.54, 18.1% ± 1.35/9.5% ± 0.32 and 23.5% ± 1.1/5.8% ± 0.88 after 12 h, 24 h and 48 h of treatment, respectively (Table 1). Similar results were found after administration of Cd and/or 5-FU for 24 h and 48 h. This effect was greater in cells treated with Cd or 5-FU/Cd compared with 5-FU alone. When cells were treated with combinations based in Cd plus 5-FU, we found decreases in the proportions of cells in the G0/G1 and G2/M phases compared with 5-FU—treated cells after 48 h of treatment (Table 1).

**Figure 1:**
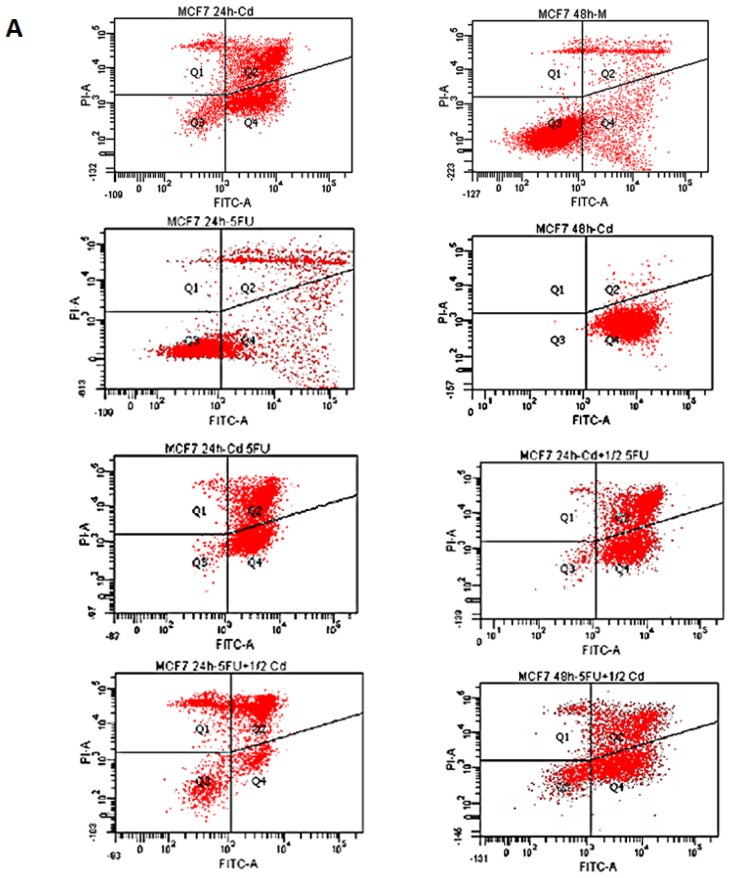

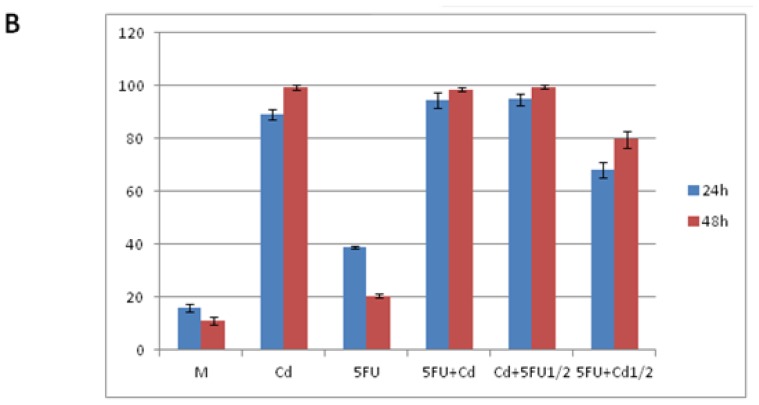
FACScan analysis via Annexin V-FITC/PI staining was used to observe the induction of apoptosis in MCF-7 cells at high doses. (**A**) Representative images of the flow cytometry analysis. Cells in the lower right quadrant indicate the percentage of Annexin-positive, early apoptotic cells. Cells in the lower left quadrant indicate the percentage of Annexin-negative/PI-negative, viable cells. Cells in the upper right quadrant indicate the percentage of Annexin-positive/PI-positive, late apoptotic cells. Cells in the upper left quadrant indicate the percentage of PI-positive, necrotic cells; (**B**) Graphic representation of apoptotic levels (early plus late apoptosis) after treatment with Cd and/or 5-FU for 24 and 48 h. Data are expressed as mean ± SEM of three independent experiments. Q, Quadrant.

For the study of the apoptosis induction we used high concentrations of Cd (5 μM) and 5-FU (3 μM) as previously reported [26,28–30]. The annexin V-FITC assay revealed that treatment with high concentrations of Cd and/or 5-FU for 24 h and 48 h potently induced apoptosis at each of the doses tested in comparison with mock-treated cells (*p* < 0.001). MCF-7 cells treated with 5 μM Cd alone showed very high apoptosis levels after 24 and 48 h of treatment (87.5% ± 3.2% and 99.9% ± 0.04%, respectively; *p* = 0.0026). Exposure to 3 μM 5-FU alone was associated with lower rates of apoptosis at 24 and 48 h (38.5% ± 0.55% and 20.2% ± 0.79%, respectively; *p* = 0.0001), which was significantly increased when Cd was added (Figure 1), except in the condition of 5-FU plus Cd added only at the half time from the experiment started (5FU½Cd) where this increase was not statistically significantly (70.1% ± 3.02% and 81.9% ± 2.34%, respectively; *p* = 0.0059) (Figure 1).

### 2.2. Gene Expression

**Figure 2:**
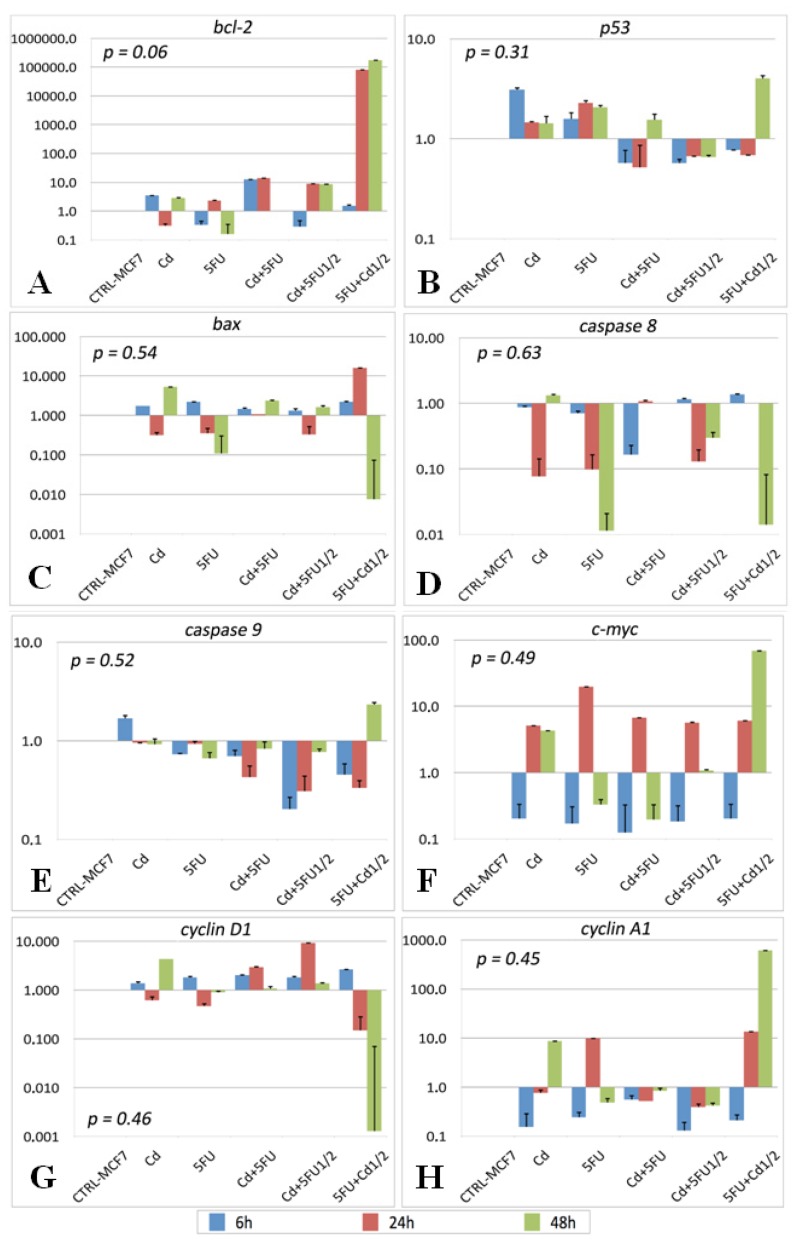
Determination by qRT-PCR of gene expression after treatment with Cd and/or 5-FU for 6, 24, or 48 h. The fold-increase in expression was quantified after normalizing expression levels for those in control MCF-7 cells. Gene expression of *bcl-2* (**A**); *p53* (**B**); *bax* (**C**); *caspase 8* (**D**); *caspase 9* (**E**); *c-myc* (**F**); *cyclin D1* (**G**); *cyclin A1* (**H**).

Gene expression was determined by qRT-PCR and the fold-increase in expression was quantified after normalizing expression levels for those in control MCF-7 cells, which were assigned the expression level of one. The *bcl2* gene was mainly upregulated in conditions where Cd was used. In cells treated with Cd alone, *bcl2* expression was increased by 0.3–2.5 times compared with control cells. The combinations of Cd plus 5-FU½ or Cd plus 5-FU increased the expression of *bcl2* by 8 and 13 times, respectively. Surprisingly, *bcl2* expression was markedly increased by treatment with 5-FU plus Cd½ for 24 and 48 h (*RFI*: 80 × 10^3^ and 173 × 10^3^, respectively). By contrast, treatment with 5-FU alone decreased *bcl2* expression at 48 h (Figure 2A).

The expression of *p53* was higher in cells treated with Cd than in control cells at 6 h (*RFI*: 2) but was 0.5 times higher at both 24 and 48 h. Its expression in cells treated with 5-FU was 0.6 times higher at 6 h, and about 2 times higher at 24 and 48 h compared with control cells. By contrast, Cd plus 5-FU and Cd plus 5-FU½ decreased *p53* expression at 6 and 24 h, but increased its expression at 48 h. Interestingly, Cd increased *p53* expression by three times in cells pre-treated with 5-FU. Cd plus 5-FU½ decreased the expression of *p53* compared with control cells (Figure 2B). The expression of *bax* was decreased in most of the experimental conditions and at most times, except for Cd at 48 h and 5-FU plus Cd½ at 24 h, in which *bax* expression was increased by 4 and 15 times, respectively. The greatest decrease in *bax* expression occurred in cells treated with 5-FU plus Cd½ for 48 h (Figure 2C). The expression levels of *caspase 8* and *caspase 9* were decreased in most of the experimental conditions and at all times (Figure 2D,E). The expression of *c-myc* was increased in all experimental conditions at 24 h, and at 48 h in cells treated with Cd or 5-FU plus Cd½ by up to 67.6 times (Figure 2F).

The gene expression levels of *cyclin D1* and *cyclin A1* were inversely correlated with each other whenever cells were treated with 5-FU plus Cd. *Cyclin D1* expression was significantly decreased and that of *cyclin A1* was increased in cells treated with 5-FU plus Cd½ for 24–48 h. Cd and 5-FU alone increased the expression levels of *cyclin A1* by up to 8 times at 48 and 24 h, respectively (Figure 2G,H).

### 2.3. Protein Expression

**Figure 3:**
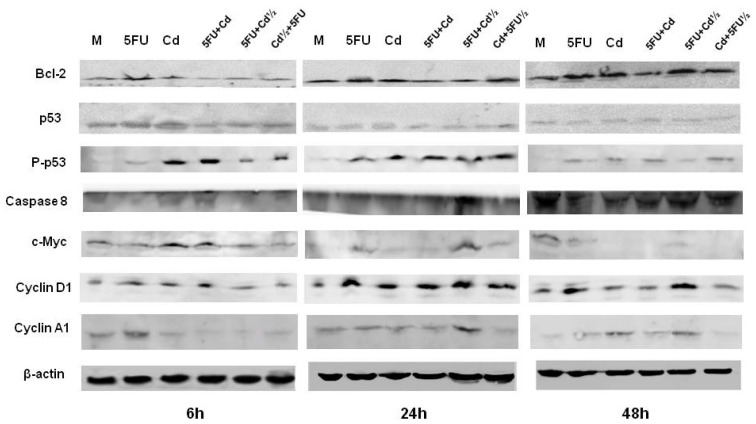
Modification of cell cycle, proliferation and apoptotic proteins analysed by western blot on the MCF-7 cell line after treatment with 5-FU and/or Cd for 6, 24 or 48 h. Representative images of three experiments.

Bcl-2 protein expression increased from 6 to 48 h in cells treated with Cd, 5-FU, and Cd plus 5-FU½. The increase in bcl-2 protein expression was particularly marked in cells treated with 5-FU plus Cd½ for 48 h (Figure 3).

Total p53 protein expression was unaffected by most of the treatments at each of the times, except in cells treated with Cd for 6 h, which increased p53 expression. However, the expression of p-p53 was markedly increased by treatment with Cd alone or in combination with 5-FU. 5-FU alone also increased p-p53 expression at 24 h (Figure 3). The protein expression of caspase 8 was unaffected in the early phase of treatment compared with control cells, except for 5-FU plus Cd½, which increased protein expression at 24 h. At 48 h, caspase 8 expression was much lower in treated cells than in control cells. However, caspase 8 expression was increased in cells treated with 5-FU plus Cd½ or Cd plus 5-FU½ (Figure 3).

Cd and 5-FU plus Cd induced c-myc expression after 6 h. Similarly, 5-FU plus Cd½ induced a significant increase in c-myc expression after 24 h. However, its expression was markedly decreased at 48 h by all treatments compared with control cells (Figure 3).

Cyclin D1 protein expression was increased by 5-FU and/or Cd at each time, with marked increases in expression in cells treated with 5-FU or 5-FU plus Cd½ for 24 and 48 h. Treatment with 5-FU and 5-FU plus Cd½ increased cyclin A1 expression at 6 and 24 h, respectively. Moreover, all of the treatment conditions increase the expression of cyclin A1, with marked increases in cells treated with Cd or 5-FU alone, or with 5-FU plus Cd½ (Figure 3).

## 3. Discussion

Epidemiological studies have suggested a link between Cd and breast cancer, but more experimental and epidemiological studies are required to establish a cause-and-effect association between exposure to Cd and the development of breast cancer [15–17]. It was previously reported that Cd increases the proliferation of MCF-7 cells and can interfere with normal cellular homeostasis, triggering signals that are otherwise turned off [28,31]. In our previous morphological and immunohistochemistry study, we reported that Cd inhibits the toxic effects of 5-FU pre-treatment on tumour cells [28]. In the present study, we performed detailed molecular analyses to evaluate the deleterious effects of Cd on the efficacy of 5-FU-based therapy.

Cd can affect cell proliferation and differentiation, cell cycle progression, DNA synthesis and repair, apoptosis, and other cellular activities [32]. In cancer cells, Cd was found to decrease the proportion of cells in the G2/M phase, and induce apoptosis, resulting in a substantial decrease in the number of viable cells [33,34]. Several studies have revealed that the cellular damage induced by 5-FU involves a loss or accumulation of cells in the S phase, G2/M block, and G1/S arrest [35]. There is some evidence suggesting that the S-phase checkpoint pathways respond to 5-FU and thymidylate synthase (TS) inhibition, and that TS inhibition and incorporation of the fluorinated base into DNA occurs during the S phase [36]. Similar results were found in the present study, as Cd and/or 5-FU decreased the proportion of cells in the G2/M phase and increased the proportion of apoptotic cells. By contrast, 5-FU plus Cd½ did not increase the proportion of apoptotic cells, which suggests that apoptosis was not markedly induced by this combination. It has been suggested that Cd-induced apoptosis might not fully protect against malignant transformation, as only a fraction of exposed cells undergo apoptosis, while the remaining cells may become resistant to apoptosis [37,38].

Apoptosis occurs via death receptor-dependent (extrinsic) or mitochondrial (intrinsic) pathways. The extrinsic pathway is triggered by the activation of death receptors, such as Fas and TRAIL (DR4, DR5), which activate the initiator caspase 8 followed by the cleavage of the executioner caspase 3. The mitochondrial route is activated in response to various internal or external stimuli, which cause a change in mitochondrial permeability. The mitochondrial pathway is regulated by members of the bcl-2 family of proteins, especially by the bax/bcl-2 ratio, which is under the control of p53. Disruption of the mitochondrial membrane potential results in the release of pro-apoptotic factors, such as cytochrome c, from the mitochondria into the cytosol, which activate caspase 9 and then caspase 3. Caspase 3 catalyses the degradation of proteins involved in vital cellular processes [30,39]. To determine whether the bax/bcl-2 pathway is involved in the responses to 5-FU or Cd, we examined the changes in these pro- and anti-apoptotic genes in the presence of Cd and/or 5-FU. Our studies in MCF-7 cells showed that Cd decreased *bax* gene expression in cells pre-treated with 5-FU, with the lowest expression level in cells treated with 5-FU plus Cd½ for 48 h. Treatment with Cd plus 5-FU increased the gene expression of *bcl-2*. Of note, treatment with 5-FU plus Cd½ for 24 and 48 h increased *bcl-2* gene expression by 80 × 10^3^ and 173 × 10^3^ times, respectively. In cells treated with 5-FU alone, *bcl-2* gene expression was decreased at 48 h, an effect that is thought to augment drug-induced apoptosis [40]. These effects of 5-FU on *bcl-2* and *bax* gene expression levels are similar to those reported by Magné *et al.* [30], who found that treatment with ZD1839 and cisplatin plus 5-FU for 24 h induced apoptosis via the mitochondrial pathway in CAL33 cells (a human head and neck cancer cell line). Furthermore, the gene expression of *caspase 9* was hardly affected by 5-FU or Cd alone, but its expression was decreased by combinations of 5-FU and Cd, similar to *caspase 8*. The reversed *bax/bcl-2* ratio and the decrease in *caspase 9* gene expression levels in cells treated with Cd exposure reflect the ability of Cd to suppress the intrinsic apoptotic pathway, which is consistent with the decreased level of apoptosis.

A similar trend was observed for the relative levels of mRNA and protein expression of p53 that was increased in cells treated with Cd for short periods of time [41], and in cells treated with 5-FU only. However, its expression was weaker in cells treated with Cd plus 5-FU. The high frequency of alterations in the p53 pathway in cancer cells underscores the importance of p53 in tumour suppression [42,43]. The effects of 5-FU on p53 expression are similar to those observed after tumour regression *in vivo*, demonstrating the therapeutic potential of reactivating p53 in established tumours [44–46]. The low expression of p53 in cells treated with Cd plus 5-FU suggests that Cd blocks the effects of 5-FU.

The protein c-myc seems to be at the crossroads of many important biological pathways and processes involved in neoplastic cell growth and proliferation. It has been shown that c-myc is broadly involved in many cancers, as its expression is either increased or disturbed in up to 70% of human cancers [47]. Elevated *c-myc* expression is associated with aggressive human prostate cancer and triple-negative breast cancer [48,49]. The gene expression profile was consistent with the protein expression profile. Cd induced marked increases in the gene and protein expression levels of the anti-apoptotic molecules bcl-2, cyclin A1, and c-myc in cells pre-treated with 5-FU. We found that the gene expression of *c-myc* was increased slightly by all treatments at 24 and 48 h. However, its expression was increased by as much as 67.6 times in cells treated with 5-FU plus Cd½. These results suggest that Cd blocks the effects of 5-FU and may increase tumour malignancy. c-myc was reported to transactivate the *cyclin A1* promoter and may be responsible for the elevated expression of *cyclin A1* in acute myeloid leukaemia [50]. Our results support this hypothesis because we observed high levels of cyclin A1 in MCF-7 cells in all treatments. Cyclin A1 plays an important role in enhanced cell proliferation in non-small cell lung cancer [50]. Moreover, *cyclin A1* mRNA and its protein are present at very low levels in cells in the G0 phase. However, these levels increase during the progression of the cell cycle, reaching the highest levels in the S and G2/M phases [51]. Cyclin D1 is a key regulatory protein that promotes the transition through the restriction point in the G1 phase [52]. In our studies, Cd plus 5-FU induced an increase in *cyclin D1* and *cyclin A1* gene and protein expression levels, consistent with the results of the cell cycle analysis.

## 4. Experimental Section

### 4.1. Cell Culture

MCF-7 cells were cultivated in Dulbecco’s modified Eagle’s medium (DMEM; Gibco, Carlsbad, CA, USA) supplemented with 10% foetal bovine serum (FBS) (Gibco, Carlsbad, CA, USA), 2.0 mmol/L glutamine, 100 U/mL penicillin, and 100 μg/mL streptomycin. Cells were grown at 37 °C in an atmosphere containing 5% CO_2_. Cells were expanded for several days until confluence in T75 flasks. The cells were trypsinised and were plated in 24-well multiplates.

### 4.2. Drugs

5-FU and CdCl_2_ were purchased from Sigma-Aldrich (St. Louis, MO, USA). For each experiment, the stock solutions were diluted in medium to the desired concentrations. The experimental conditions are listed in Table 2. For treatments, we used concentrations ranged from 3 to 5 μM for Cd and 1.5 to 3 μM for 5-FU as previously described [28].

### 4.3. Cell Cycle Distribution Analysis

The cells at 70% confluence were treated with Cd and/or 5-FU. After 6, 12, 24, and 48 h of treatment, fluorescence-activated cell sorting (FACS) analysis was performed as previously described [53]. Cells in exponential growth were plated on 6 well plates (5 × 10^3^ cells/well) and were placed in an incubator overnight. After treatment, the cells were harvested, washed twice with phosphate-buffered saline (PBS), and fixed in 70% (*v*/*v*) cold ethanol for up to 1 week. After centrifuging the cells, the pellet was washed once with PBS and resuspended in 250 μL of propidium iodide (PI) solution (100 μL/mL RNAsa, 40 μL/mL PI in PBS) for 30 min in the dark at 37 °C. The samples were immediately analysed using a FACS can flow cytometer at the Scientific Instruments Centre (University of Granada, Granada, Spain).

### 4.4. Apoptosis Detection by Staining with Annexin V-FITC and Propidium Iodide

The annexin V-FITC apoptosis detection kit I (Pharmingen, San Diego, CA, USA) was used to determine the number of apoptotic cells by flow cytometry, as previously described [28]. Briefly, cells were plated in six well plates and were placed in the incubator overnight. Cells were then treated with high concentrations of Cd (5 μM) and/or 5-FU (3 μM). After 24 and 48 h of treatment, the cells were trypsinised and analysed using the Annexin V–FITC kit. The samples were immediately processed by Becton Dickinson FACSAria III flow cytometry at the Scientific Instruments Centre (University of Granada).

### 4.5. Gene Expression

After treatment with Cd and/or 5-FU for 2, 6, 24, or 48 h, RNA was extracted from the cells using Trizol reagent (Invitrogen, Carlsbad, CA, USA). RNA (1 μg) was reverse transcribed and the resulting cDNA was subjected to quantitative real-time polymerase chain reaction (qRT-PCR) to determine the expression of specific genes (*bcl2*, *bax*, *caspase 8*, c*-myc*, *cyclin A1*, and *cyclin D1*; Table 3) involved in growth and proliferation of MCF-7 cells. qRT-PCR was performed using SYBR^®^ Green (Invitrogen, Carlsbad, NM, USA) on an iCycler with version 2.0 software (iQ; Bio-Rad, Hercules, CA, USA). The reaction mixture (total volume, 50 μL) comprised 25 μL SYBR Mix, 2 μL of each of the forward and reverse primers (10 pmol/μL; final concentration, 400 nM), and 5 μL of cDNA. Overall, 50 cycles were performed, with each amplification cycle consisting of denaturation at 94 °C for 15 s, 55 °C for 30 s, and 60 °C for 30 s, after which fluorescence was measured. All primers were purchased from Invitrogen. The cycle threshold (Ct) values were determined for the amplification of *bcl2*, *bax*, *caspase 8*, *c-myc*, *cyclin D1*, *cyclin A1*, and *GAPDH*, and *ΔCt* was calculated by subtracting the *Ct* value for *GAPDH* from the *Ct* value for each target gene. Expression of the target genes was normalized according to that of *GAPDH*. The relative fold increase (*RFI*) was calculated by first determining the *ΔCt* for treated and control cells using the following equation: *ΔCt* = *Ct* (gene) − *Ct* (*GAPDH*). The *ΔΔCt* value was then determined by subtracting the *ΔCt* value for the treated cells from the *ΔCt* value for the control cells, and was used to calculate the *RFI* for the target gene using the following equation: *RFI* = 2 − *ΔΔCt*.

### 4.6. Protein Expression

Cells were plated in 6-well plates in DMEM supplemented with 10% foetal bovine serum (FBS) (Gibco, Carlsbad, CA, USA), 2.0 mmol/L glutamine, 100 U/mL penicillin, and 100 μg/mL streptomycin. After 24 h, the cells were induced with 5-FU and/or Cd for 6, 24 or 48 h. Parallel cultures lacking 5-FU or Cd were used as controls. At the indicated times, the medium was removed and cells were lysed in lysis buffer (60 mMTris/HCl pH 6.8, 25% glycerol, 2% sodium dodecyl sulphate (SDS), 14.4 mM 2-mercaptoethanol and 0.1% bromophenol blue). The lysed cells were stored at −20 °C until use. To assess protein expression, cells were thawed and boiled for 10 min at 96 °C. Protein samples (20 μg) were then subjected to SDS—polyacrylamide gel electrophoresis in a Mini Protean II cell (Bio-Rad, Hercules, CA, USA) at 60 mA for 3 h at room temperature. The proteins were then transferred to a nitrocellulose membrane by applying a current of 20 V for 45 min at room temperature. To verify protein transfer, the nitrocellulose membrane was stained with Ponceau, and then washed twice with PBS for 10 min each. The membranes were treated with blocking solution (5% non-fat milk in PBS) for 1 h at room temperature and washed three times with PBS for 10 min each. The membranes were then incubated with the primary antibody and diluted in 5% non-fat milk in PBS, overnight at 4 °C with agitation. After washing three times with PBS, the membranes were incubated with the secondary antibody for 1 h, washed, and bands were visualized using an enhanced chemiluminescent system (Amersham Pharmacia Biotech, Little Chalfont, UK). Primary antibodies for bcl2, p53, phosphorylated (p)-p53, caspase8, c-myc, cyclin D1, and cyclin A1 were from Santa Cruz (Santa Cruz, CA, USA). A monoclonal antibody against β-actin (A2228) and the secondary antibodies (horseradish peroxidase-conjugated anti-rabbit IgG (A0545) and horseradish peroxidase-conjugated anti-mouse IgG (A9044]) were from Sigma-Aldrich (St. Louis, MO, USA).

### 4.7. Statistical Analysis

Statistical analysis was performed using the STATA SE12 statistical program [54]. The determination by qRT-PCR of gene expression after treatment with Cd and/or 5-FU that were measured over time was analyzed by ANOVA analysis. For comparisons of all proportion means, the Student *t* test was used. In all cases, *p* ≤ 0.05 was taken to be significant.

## 5. Conclusions

In the present study, and based on prior reports, we established an *in vitro* model to reproduce the conditions of patients with breast cancer treated with 5-FU with environmental exposure to Cd, as a representative heavy metal. Our results have improved our understanding of the potential effects of Cd on cell physiology, as well as the possible implications for the development of breast cancer. Our results suggest that the efficacy of 5-FU could be reduced in patients with breast cancer who have been already been exposed to Cd, as Cd inhibits the cytotoxicity of 5-FU and hence decreases its effectiveness. Chronic exposure to Cd is a risk factor for the development of cancer and must be taken into account when choosing the chemotherapeutic regimen. Further *in vivo* studies are needed to elucidate the exact effects of Cd exposure on the efficacy of chemotherapies. We believe that our findings provide a foundation for performing such studies in the future, especially in patients with breast cancer under chemotherapy and who are exposed to Cd from the environment or from passive/active smoking.

## Figures and Tables

**Table 1:** Cell cycle distribution induction in the MCF-7 human breast cancer cell line after treatment for 6, 12, 24 or 48 h. Data are expressed as mean of % ± SEM of three independent experiments.

	M	Cd	5FU	5FU + Cd	Cd + 5FU½	5FU + Cd½
**6 h**						

**G0/G1**	56.7 ± 1.2	61.1 ± 1.64	59.0 ± 0.85	61.5 ± 1.23	62.9 ± 0.86	61.1 ± 2.34
**S**	28.0 ± 0.95	23.0 ± 1.06	28.6 ± 0.19	24.2 ± 1.27	24.9 ± 0.56	26.8 ± 1.21
**G2/M**	14.0 ± 0.35	15.8 ± 0.57	12.2 ± 0.78	14.2 ± 0.19	11.3 ± 0.75	15.7 ± 0.1

**12 h**						

**G0/G1**	61.6 ± 2.07 *	49.0 ± 1.19 **	62.1 ± 1.27 *	58.9 ± 1.21	61.8 ± 0.93	60.0 ± 1.54
**S**	21.7 ± 1.54 *	26.1 ± 0.56 *	24.6 ± 0.29 ***	26.2 ± 0.31	22.2 ± 1.07 *	26.9 ± 0.6
**G2/M**	16.2 ± 0.77 *	6.9 ± 0.74 *	12.9 ± 0.35	14.4 ± 0.43	14.6 ± 0.65 *	12.6 ± 0.22 ***

**24 h**						

**G0/G1**	81.7 ± 2.88 **	66.9 ± 1.2 *	75.2 ± 2.33 **	63.0 ± 1.54	74.9 ± 1.76 **	72.2 ± 2.81
**S**	9.5 ± 0.32 ***	18.1 ± 1.35 *	15.3 ± 0.88 ***	15.7 ± 1.02 **	14.0 ± 0.58 ***	15.2 ± 0.76 **
**G2/M**	8.5 ± 0.08 ***	5.5 ± 0.1 **	8.8 ± 0.55 *	2.3 ± 0.12 ***	3.1 ± 0.05 ***	9.5 ± 0.56 ***

**48 h**						

**G0/G1**	85.9 ± 3.21 **	60.5 ± 2.03	82.9 ± 3.08 **	72.3 ± 2.44 *	75.5 ± 1.56 **	78.2 ± 1.8 **
**S**	5.8 ± 0.88 ***	23.5 ± 1.1	9.2 ± 0.77 ***	11.5 ± 1.1 **	12.3 ± 0.9 ***	9.0 ± 1.3 **
**G2/M**	6.7 ± 0.5 ***	12.3 ± 0.21 **	4.9 ± 0.4 **	0.3 ± 0.02 ***	0.2 ± 0.01 ***	0.9 ± 0.03 ***

M: mock, control non-treated cells; 5FU + Cd: cells treated with both drugs; Cd + 5FU½: cells treated with Cd plus 5-FU added after the half of time from the experiment started; 5FU + Cd½: cells treated with 5-FU plus Cd added after the half time from the experiment started. Difference of mean value of proportion of cells in each cell cycle was tested using Student *t* test, considering 6 h after treatment as reference for the comparison;

* *p* < 0.05;

** *p* < 0.001;

*** *p* < 0.0001.

**Table 2:** MCF-7 experimental conditions.

		Time points
**M**	Control non treated cells	6 h, 24 h, 48 h
**Cd**	Cells treated with Cd	6 h, 24 h, 48 h
**5FU**	Cells treated with 5FU	6 h, 24 h, 48 h
**Cd + 5FU**	Cells treated with both drugs	6 h, 24 h, 48 h
**Cd + 5FU_½_**	Cells treated with Cd plus 5-FU added after the half of time from the experiment started	6 h, 24 h, 48 h
**5FU + Cd_½_**	Cells treated with 5-FU plus Cd added after the half time from the experiment started	6 h, 24 h, 48 h

**Table 3:** Primers used for qRT-PCR.

	Primer	Annealing temperature (°C)
***bcl-2***	Forward: 5′-TGGTGGTTTGACCTTTAGAGA-3′ Reverse: 5′-AGGTCTGATCATTCTGTTC-3′	55
***p 53***	Forward: 5′-GGCATTCTGGGAGCTTCATCT-3′ Reverse: 5′-CCCAAGCAATGGATGATTTGA-3′	58.5
***bax***	Forward: 5′-TGCTTCAGGGTTTCATCCAG-3′ Reverse: 5′-GGCGGCAATCATCCTCTG-3′	55
***caspase 8***	Forward: 5′-AGGAGGAGATGGAAAGGGAACTT-3′ Reverse: 5′-ACCTCAATTCTGATCTGCTCACTTCT-3′	55
***caspase 9***	Forward: 5′-CCTCAAACTCTCAAGAGCAC-3′ Reverse: 5′-GAGTCAGGCTCTTCCTTTG-3′	58.5
***c-myc***	Forward: 5′-GGACGACGAGACCTTCATCAA-3′ Reverse: 5′-CCAGCTTCTCTGAGACGAGCTT-3′	55
***cyclin D1***	Forward: 5′-CCGTCCATGCGGAAGATC-3′ Reverse: 5′-ATGGCCAGCGGGAAGAC-3′	55
***cyclin A1***:	Forward: 5′-GCACCCTGCTCGTCACTTG-3′ Reverse: 5′-AGCCCCCAATAAAAGATCCAG-3′	55
***GAPDH***	Forward: 5′-CAAGGAGTAAGACCCCTGGAC-3′ Reverse: 5′-TCTACATGGCAACTGTGAGGAG-3′	58.5
